# Natural *Clerodendrum*-derived tick repellent: learning from Nepali culture

**DOI:** 10.1007/s10493-023-00804-4

**Published:** 2023-06-07

**Authors:** Lorena Mazuecos, Marinela Contreras, Paul D. Kasaija, Prajwol Manandhar, Weronika Grąźlewska, Eduardo Guisantes-Batan, Sergio Gomez-Alonso, Karelia Deulofeu, Isabel Fernandez-Moratalla, Rajesh Man Rajbhandari, Daniel Sojka, Libor Grubhoffer, Dibesh Karmacharya, Christian Gortazar, José de la Fuente

**Affiliations:** 1 Instituto de Investigación en Recursos Cinegéticos IREC-CSIC-UCLM-JCCM, Ronda de Toledo s/n, Ciudad Real, 13005 Spain; 2 National Livestock Resources Research Institute (NaLIRRI/NARO), Wakiso District, P.O. Box 5704, Wakiso, Uganda; 3 Center for Molecular Dynamics Nepal (CMDN), Thapathali Road 11, Kathmandu, 44600 Nepal; 4 Department of Molecular Biotechnology and Microbiology, Faculty of Chemistry, Gdańsk University of Technology, Gdańsk, 80-233 Poland; 5 Instituto Regional de Investigación Científica Aplicada (IRICA), Universidad de Castilla-La Mancha, Ciudad Real, 13005 Spain; 6 KGJ Collection, Adelfa 75, Ciudad Real, 13005 Spain; 7 7 Manantiales 2, Villanueva de los Infantes, 13320 Spain; 8 Institute of Parasitology, Biology Centre, Academy of Sciences of the Czech Republic, Branišovská 1160/31, České Budějovice, 37005 Czech Republic; 9 Department of Veterinary Pathobiology, Center for Veterinary Health Sciences, Oklahoma State University, Stillwater, OK 74078 USA

**Keywords:** Tick, Repellent, *Clerodendrum*, Otoacariasis, Nepal

## Abstract

Ticks attaching to ear canals of humans and animals are the cause of otoacariasis, common in rural areas of Nepal. The plant *Clerodendrum viscosum* is used in multiple indigenous systems of medicine by ethnic communities in the Indo-Nepali-Malaysian region. Visiting the Chitwan National Park, we learned that in indigenous medicine, flower extract of *C. viscosum* is utilized to treat digestive disorders and extracts from leaves as tick repellent to prevent ticks from invading or to remove them from the ear canal. The objective of our study was to provide support to indigenous medicine by characterizing the in vivo effect of leave extracts on ticks under laboratory conditions and its phytochemical composition. We collected plant parts of *C. viscosum* (leaves and flowers) and mango (*Mangifera indica*) leaves at the Chitwan National Park, previously associated with repellent activity to characterize their effect on *Ixodes ricinus* ticks by in vivo bioassays. A Q-ToF high-resolution analysis (HPLC-ESI-QToF) was conducted to elucidate phenolic compounds with potential repellent activity. *Clerodendrum viscosum* and *M. indica* leaf extracts had the highest tick repellent efficacy (%E = 80–100%) with significant differences when compared to *C. viscosum* flowers extracts (%E = 20–60%) and phosphate-buffered saline. Phytochemicals with tick repellent function as caffeic acid, fumaric acid and p-coumaric acid glucoside were identified in *C. viscosum* leaf extracts by HPLC-ESI-QToF, but not in non-repellent flower extracts. These results support the Nepali indigenous medicine application of *C. viscosum* leaf extracts to repel ticks. Additional research is needed for the development of natural and green repellent formulations to reduce the risks associated with ticks resistant to acaricides.

**Supplementary Information:**

The online version contains supplementary material available at 10.1007/s10493-023-00804-4.

## Introduction

Ticks of genera such as *Amblyomma*, *Dermacentor*, *Haemaphysalis*, *Hyalomma*, *Ixodes*, and *Rhipicephalus* are widespread throughout Nepal including the Chitwan National Park with associated health risks for humans and wildlife (Pun et al. 2018). Intra-aural ticks attached to the external auditory canal may cause peripheral facial nerve paralysis (Doğan et al. 2012; Kularatne et al. 2018). Ticks and (other) mites attached within the ear canal are the cause of otoacariasis common in rural areas of Nepal and other countries in both humans and animals, making ectoparasite removal hard with partially efficient treatment interventions (Fegan and Glennon 1996; Indudharan et al. 1999; Somayaji and Rajeshwari 2007; Doğan et al. 2012; Cakabay et al. 2016; Kwak et al. 2021). Due to culture and economic resources, rural Asiatic areas have developed traditional methods for control of local parasites or food preservation (Salehi et al. 2018; Riaz et al. 2020). Indeed, research on these methods may provide new alternatives for ‘green’ chemical formulations. It has been recently reported that some compounds synthetically derived from botanical sources have acaricidal activity against *Ixodes scapularis* similar to repellents in the market, supporting a scientific base to traditional and natural medicine and arising new perspective for green chemical-based strategies (Lee et al. 2022).

The plant *Clerodendrum viscosum* Vent (Lamiaceae; synonym *Clerodendrum infortunatum* L.; hill glory bower or Bhatti in Nepali) germinates in June-July and flowers in February-March, and is distributed throughout tropical and subtropical regions of Asia, Africa and the Pacific with particular relevance in the Indo-Nepali-Malaysian region (Srivastava et al. 2021). Together with other natural resources, *C. viscosum* has been described as a traditional botanical medicine and is the primary mode of health care by local medical experts in indigenous systems of Nepal and Pakistan communities (Bhattarai et al. 2010; Ishtiaq et al. 2021). In these rural areas, *C. viscosum* is used as an effective tool for human health against cough/cold, itching, indigestion and abdominal pain (Bhattacharjee and Ray 2010). In the Unani medical system, leaf decoction has been applicated in rheumatism system and seed power as vermicide treatment (Singh et al. 1997). *Clerodendrum viscosum* plant extracts and their phytoconstituents have shown anti-inflammatory, antioxidant, antidiabetic, anticancer, immunomodulatory, hemagglutination, antimicrobial, insecticidal and hepatoprotection pharmacological properties under laboratory conditions (Bird 1961; Luitel et al. 2014; Nandi and Lyndem 2016; Ishtiaq et al. 2021; Shendge et al. 2021a, b; Srivastava et al. 2021) and used in asthma, malaria or blood and respiratory system diseases (Nandi and Lyndem 2016; Joshi et al. 2020). A gas chromatography-mass spectrometry (GC-MS) analysis on methanol extract of *C. viscosum* demonstrated the presence of steroids, triterpenoids and flavonoids (Ghosh et al. 2015). The two major bioactive compounds found by Ghosh et al. (2015) are N,N-dimethylglycine and 3-deoxy-d-mannoic lactone previously reported to have immune modulating properties and antibacterial activity (Graber et al. 1981). In vitro studies have shown apoptotic activity of bioflavonoid apigenin isolated from *Clerodendrum* leaves (Shendge et al. 2017, 2021a) and anticancer activity of 70% methanolic extracts (Shendge et al. 2021a). However, there is no information for its potential as tick repellent.

Natural repellents are an approach to decrease and overcome the risks associated with tick acaricide resistance and for integrated control of ticks and tick-borne diseases (de la Fuente 2018; Quadros et al. 2020; Wang et al. 2022; Sharma et al. 2022). The main phytochemicals identified in *C. viscosum* are monoterpenoids, diterpenoids, triterpenoids, glycosides, saponins, steroids, and flavonoids (Nandi and Lyndem 2016; Quadros et al. 2020; Srivastava et al. 2021). Plant-derived alkaloids, monoterpenoids such as thymol, carvacrol and linalool and essential oils diterpenoids have been characterized as potential acaricide with repellent and toxic activity against ticks (Gross et al. 2017; Schubert et al. 2017; Tabari et al. 2017; Adenubi et al. 2018a; Soutar et al. 2019; Nwanade et al. 2020; Quadros et al. 2020; Luns et al. 2021).

**Fig. 1:**
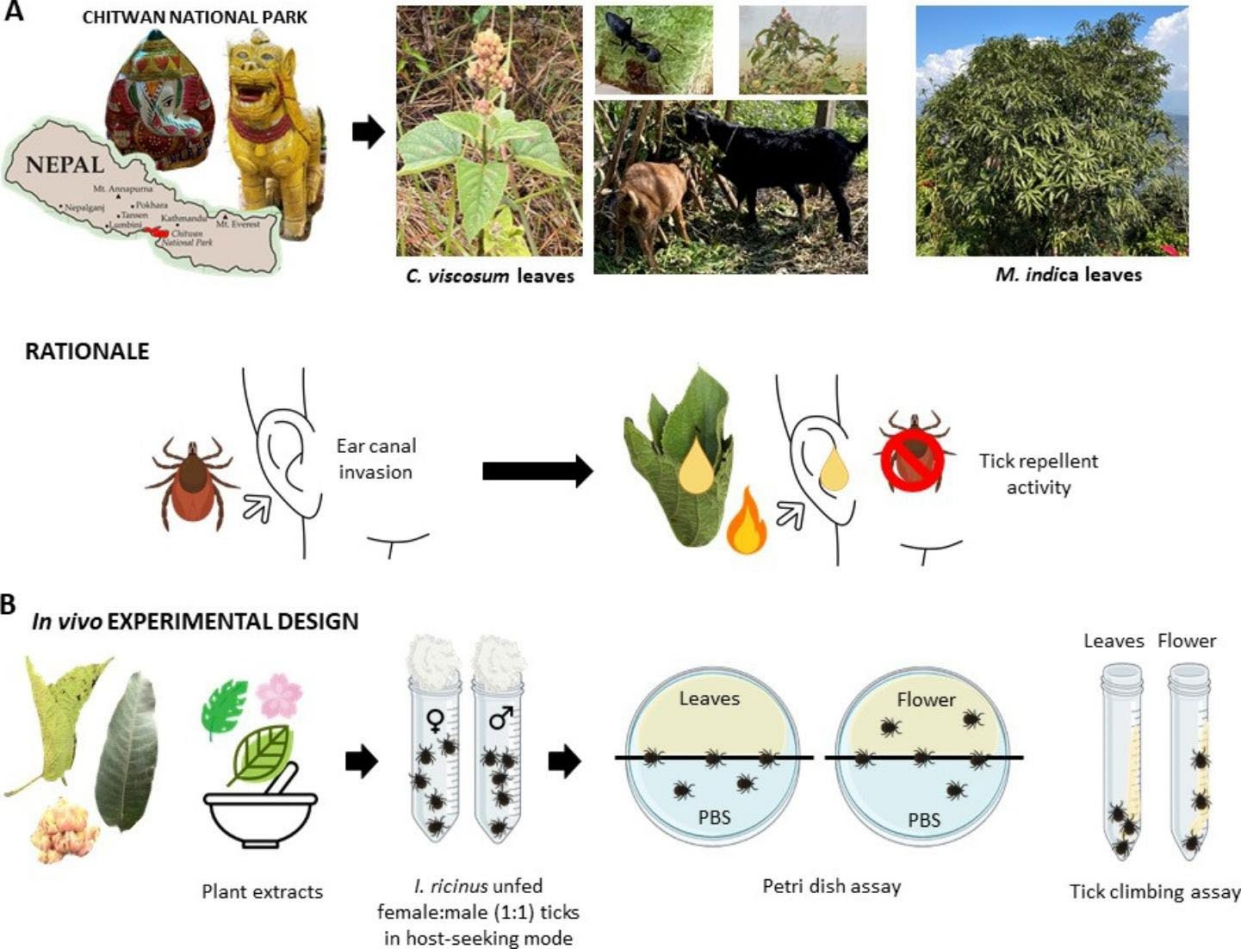
Study rationale and experimental design. **(A)** In the Nepali Chitwan National Park, indigenous medicine uses extracts from *Clerodendrum viscosum* (Bhatti) and *Mangifera indica* (mango) plant leave extracts to prevent ticks from invading or to remove them from the ear canal. Nepalis construct a funnel with leaves, place it on the ear canal and add a burning coal piece to extract plant leaf juice with repellent activity. **(B)** Based on this information, we collected at the Nepali Chitwan National Park *C. viscosum* leaves and flowers together with mango leaves also associated with repellent activity to characterize their effect on ticks under laboratory conditions. Photographs were taken on Chitwan National Park by researchers

When we visited the Chitwan National Park we learned that in Nepali indigenous medicine, extracts of *C. viscosum* plants were used for treatment of digestive disorders (flowers), hemagglutination (fruits) and tick repellent (leaves). Extracts from other *Clerodendrum* species (*Clerodendrum glabrum*, also known as *Rotheca glabrum*) were used as repellent against adults of *Rhipicephalus appendiculatus* and for the control of cattle ticks in South Africa (Mawela et al. 2019). *Clerodendrum* plants are also used in this area for feeding goats. Although insects can be found on *C. viscosum* plants, community members claim that ticks are rarely found in areas with these plants with protection to humans and wildlife (Fig. 1A). To prevent ticks from invading or to remove them from the ear canal, Nepalis construct a funnel with leaves, place it on the ear canal and add a burning coal piece to extract plant leaf juice with repellent activity (Fig. 1A). Based on this information, we collected at the Chitwan National Park Bhatti *C. viscosum* leaves and flowers together with mango (*Mangifera indica*) leaves also associated with repellent activity (Alwala et al. 2010) to characterize their in vivo effect on ticks under laboratory conditions and phytochemical composition.

## Materials and methods

### Plant extracts

Extracts from leaves and flowers of *C. viscosum* (Fig. 1A) and leaves of mango were prepared. All extracts were used to evaluate tick repellency. The plant name is recorded by The World Flora Online List (previous www.theplantlist.org), record kew-43,162. Samples were collected in February 2022 at the Nepali Chitwan National Park. To mimic conditions used by indigenous medicine to remove/repel ticks, plant leaves and flowers were separated manually, and the aqueous plant extract prepared shortly before application. Leaves or flowers were ground in 2-ml tubes with 3-mm-diameter G25 chrome steel (AISI 52,100) beads (Amazon, Ciudad Real, Spain) using a vortex mixer (Stuart Scientific, Merck, Germany) as previously described (Huynh et al. 2017). Grinded plant components were then smashed using a mortar and pestle in 5% wt/wt sterile distilled water for 10 min and then stored at room temperature overnight. The aqueous extracts were filtered using a 0.2 mm filter (Millipore, Burlington, MA, USA) to remove particulate matter. Dimethyl sulfoxide (DMSO) extracts were prepared by cutting plant samples in small pieces and smashing them with a mortar and pestle. Aliquots of 3 ml of DMSO were added during the smashing process and plant extracts were placed in agitation set at 4 °C overnight with agitation in an SB3 Stuart rotating shaker (Biolab, Barcelona, Spain) and then centrifuged at 4000× g for 10 min to collect supernatant. Negative controls were based on phosphate-buffered saline (PBS) for both aqueous and DMSO plant extracts.

### Ixodes ricinus ticks

Experimental *I. ricinus* ticks were obtained from a laboratory colony maintained at the Institute of Parasitology, Biology Centre of the Czech Academy of Sciences (BC CAS), Ceske Budejovice, Czech Republic (Hartmann et al. 2018). Same age unfed female and male ticks were provided by D. Sojka and L. Grubhoffer (CAS). Ticks were maintained in the rearing facility at CAS under controlled conditions (L12:D12 photoperiod, 24 °C and 95% r.h.). All laboratory animals were treated in accordance with the Animal Protection Law of the Czech Republic No. 246/1992 Sb., ethics approval No. 25/2018. The study was approved by the Institute of Parasitology, Biology Centre CAS and Central Committee for Animal Welfare, Czech Republic (Protocol No. 1/2015).

### Petri dish and tick climbing repellency bioassays

To evaluate tick repellent activity, plant aqueous and DMSO extracts were assayed using previously validated Petri dish and tick climbing repellency bioassays (Fig. 1B) (Dautel 2004; Gliniewicz et al. 2017; Adenubi et al. 2018b).

#### Petri dish repellency bioassay

A filter paper with an open hole (approx. 35 mm diameter) in the center was placed on the Petri dish. Petri dishes were maintained in a chamber without contact with observers at 25 °C. Plant extracts or PBS (0.5 ml) were applied to different sides of the paper and after 5 min to dry, 10 unfed *I. ricinus* ticks (1:1 female:male) under questing behavior were added in the dish open center. Ticks can either enter and remain on the surface treated with plant extract or on the control PBS-treated surface. Tick counts were recorded after 10 min. Experiments with 10 ticks each were repeated 2× for each treatment. Repellent efficacy (%E) was calculated as [(total number of ticks – number of ticks on plant extract filter side)/10] × 100%. Similar results were obtained with plant aqueous and DMSO extracts and thus used together to evaluate significant repellency by comparison between both sides of the dish and groups, using a one-way ANOVA test followed by post-hoc Tukey honestly significant difference (HSD) test to separate means, applying the Bonferroni–Holm method (α = 0.05, n = 4 biological replicates).

#### Tick climbing repellency bioassay

To provide additional support, a single trial was conducted under tick climbing repellency bioassay conditions. Plant aqueous extracts or PBS (0.4 ml) were added to filter paper strips and placed inside surface of Corning 15-ml centrifuge polypropylene, conical bottom tubes (Merck, Rahway, NJ, USA). After 5 min to dry, 10 unfed *I. ricinus* ticks (1:1 female:male) under questing behavior were added to the bottom of the tube. The ticks climbing on the filter papers and thus not repelled by treatment were counted 10 min after and the percentages of ticks climbing on were calculated and compared between groups.

#### Tick behavior

The behavior of selected ticks was recorded in the border of the filter paper between plant extracts and PBS control (Supplementary Video_1 and Supplementary Video_2).

### Q-ToF high-resolution mass spectrometry analysis (HPLC-ESI-QToF)

The identification of phenolic compounds presented in both aqueous *C. viscosum* plant extracts (flower and leaves) was carried out by HPLC Agilent 1260 system coupled to a 6545 quadrupole-time of flight (Q-ToF) mass spectrometer detector (Agilent, Waldbronn, Germany). The Q-ToF used a Dual Jet Stream Electrospray Ionization (Dual AJS-ESI) source operated in both positive and negative ionization modes following the methods previously described by Torres-Vega et al. (2020) and Bordiga et al. (2013). For the positive ionization mode, the following parameters were set: capillary voltage, 3500 V; fragmentor, 150; nozzle voltage, 1000 V; gas temperature, 350 °C; gas flow 8 l/min; nebulizer, 40 psig; sheath gas temperature, 400 °C; sheath gas flow, 10 l/min; acquisition range, 100–1200 *m*/*z*. Samples were analysed after injection of 10 µl of each extract on a Zorbax Eclipse Plus C18 Rapid Resolution HD column (2.1 × 50 mm, 1.8 μm particle size; Agilent, Santa Clara, CA, USA), thermostat at 40 °C and a flow rate of 0.3 ml/min. The solvent system was 0.1% formic acid for solvent A and 0.1% formic acid in methanol for solvent B. The elution gradient was (time, % of solvent B): 0 min, 7%; 10 min, 20%; 40 min, 75%; 46.5 min, 95%; and 56 min, 7%. For the negative ionization mode, the parameters were set: capillary voltage, 3500 V; fragmentor, 150 V; nozzle voltage, 300 V; gas temperature, 300 °C; gas flow, 11 l/min; nebulizer, 20 psig; sheath gas temperature, 350 °C; sheath gas flow 11 l/min and acquisition range, 100–1200 *m*/*z.* Samples (10 µl) were analysed into an Acentis C18 reversed phase column (150 × 4.6 mm, 2.7 μm particle size; Supelco, Darmstadt, Germany), thermostat at 16 °C with a flow rate of 0.3 ml/min. The composition of mobile phase was the same with the positive ionization mode with the employed gradient (time, % of solvent B): 0 min, 7%; 25 min, 32%; 40 min, 57%; 50 min, 67%; 55 min, 97%; 65 min, 97%; and 70 min, 7%. The control software was Mass Hunter Workstation v.B.06.11 (Agilent, Santa Clara). Compounds were identified using the algorithm ‘Find by Formula’ that evaluated the mass accuracy together with the isotopic relative abundance and isotopic separation.

### Results and discussion

**Fig. 2:**
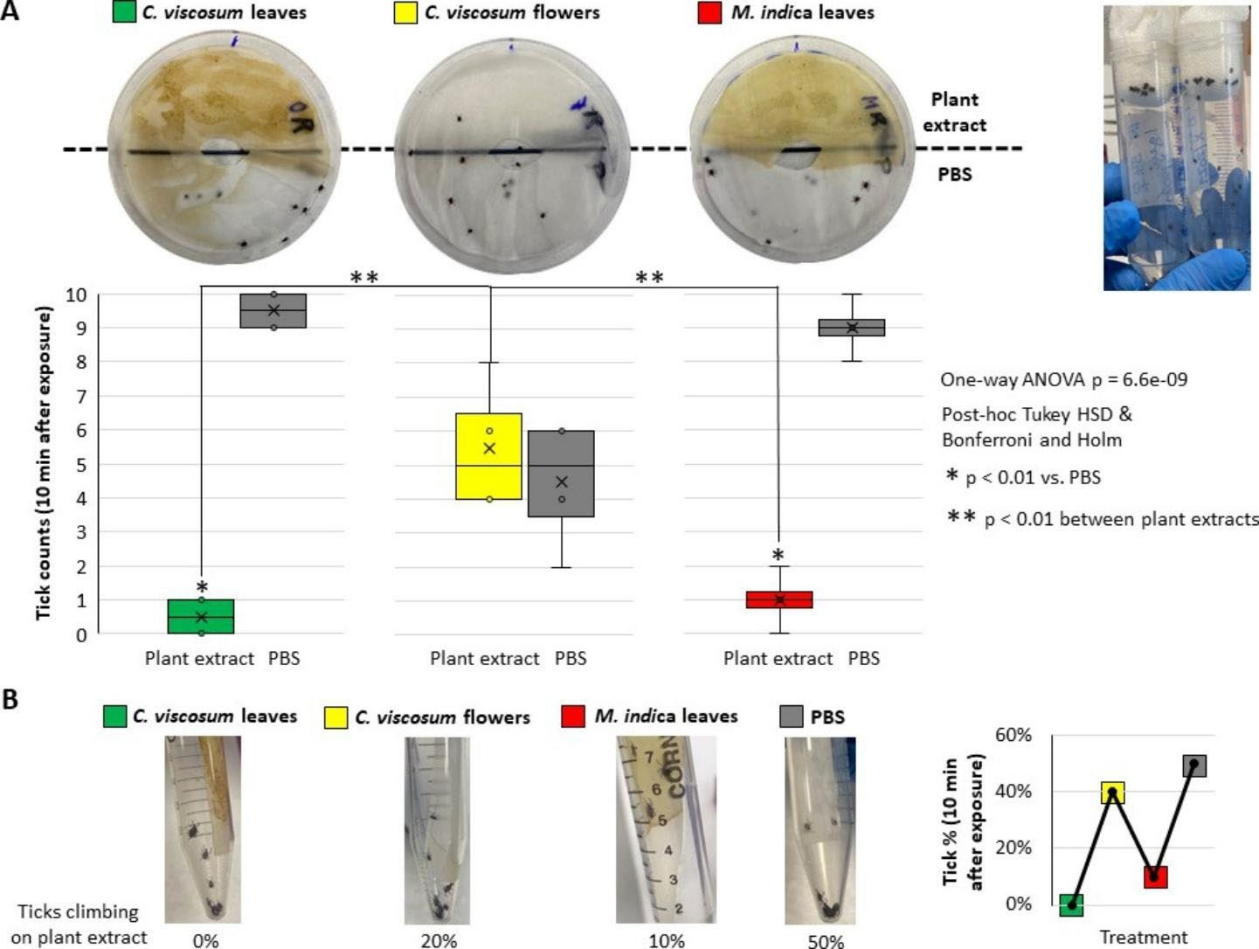
Evaluation of tick repellent activity of plant extract. Plant extracts were prepared from *Clerodendrum viscosum* (Bhatti) leaves and flowers and *Mangifera indica* (mango) leaves and used to evaluate tick repellency. (**A**) Petri dish repellency bioassay. A filter paper with an open hole in the center was placed in the Petri dish. Plant extracts or phosphate-buffered saline (PBS, control) were applied to different sides of the paper and after 5 min, 10 unfed questing *Ixodes ricinus* ticks (1:1 female:male) were added in the dish center. Tick counts after 10 min were recorded and used to evaluate repellency by comparison between both sides of the dish and groups combined by plant components (one-way ANOVA followed by Tukey HSD test and Bonferroni–Holm method; n = 4 biological replicates). (**B**) To provide additional support, a single trial was conducted under tick climbing repellency bioassay conditions. Plant extracts or PBS were added to filter paper strips and placed inside 15-ml Corning tubes. After 5 min, 10 unfed questing *I. ricinus* ticks (1:1 female:male) were added to the bottom of the tube. Ten min later, the percentage of ticks climbing on the filter papers was calculated and compared between groups

Based on Nepali Chitwan National Park indigenous medicine practice, extracts from *C. viscosum* leaves and flowers and mango leaves were bioassayed for repellency against *I. ricinus* ticks using Petri dish and tick climbing bioassay techniques (Fig. 1B). Our results showed that *C. viscosum* and *M. indica* leaf extracts repelled ticks with highest tick repellent efficacy (%E) of 80–100% (Table 1) with significant differences compared with extract from flowers of *C. viscosum* (%E = 20–60%, Table 1) and PBS (Fig. 2A).

In agreement with the indigenous medicine application of *C. viscosum* flowers for the treatment of digestive disorders but not against ticks, the tick repellency effect was not significantly different from PBS control (Fig. 2A). Similar results were obtained in the single tick climbing repellency bioassay done to provide additional support to the Petri dish repellency bioassay (Fig. 2B).

Tick behavior was recorded for selected ticks in the border of the filter paper between plant extracts and PBS control. The procedure employed and results with *C. viscosum* leaves aqueous extract and *M. indica* leaves DMSO extract showed how some ticks moved immediately away from the plant extract while others explored the possibility of crossing the border but ended moving into the PBS control side (Supplementary Videos 1 and 2.).

These results provided evidence supporting the Nepali indigenous medicine application of extracts from *C. viscosum* leaves to repel ticks from invading or to remove them from the ear canal (Fig. 1B). Based on these findings, additional research should focus on identifying the *C. viscosum* phytochemicals with tick repellent function to further explore the development of natural formulations and reduce the risks associated with ticks resistant to acaricides. To address this objective, HPLC-ESI-QToF analysis was carried out and revealed the presence of phenolic compounds in flower, leaves or both aqueous extracts from *C. viscosum* (Table 2).

**Table 1:** Tick repellent effect of *Clerodendrum viscosum* (Bhatti) and *Mangifera indica* (mango) plant extracts on Petri dish repellency bioassays

Plant extract	Tick counts on plant extract filter side	Tick counts on phosphate-buffered saline (control) filter side	Repellent efficacy (%E)	Mean (± SD) %E
*C. viscosum* leaves aqueous extract	0	10	100	95 ± 7
	1	9	90	
* C. viscosum* leaves DMSO extract	0	10	100	95 ± 7
	1	9	90	
* C. viscosum* flowers aqueous extract	4	6	60	50 ± 14
	6	4	40	
* C. viscosum* flowers DMSO extract	4	6	60	40 ± 28
	8	2	20	
*M. indica* leaves aqueous extract	1	9	90	85 ± 7
	2	8	80	
*M. indica* leaves DMSO extract	1	9	90	95 ± 7
	0	10	100	

A total of 10 ticks per treatment were used with 2 replicates. Repellent efficacy (%E) = [(total no. ticks – no. ticks on plant extract filter side)/10] × 100%. DMSO: dimethyl sulfoxide

**Table 2:** Phenolic compounds identified from *Clerodendrum viscosum* with the HPLC coupled with quadrupole time-of-flight high resolution mass spectrometry (HPCL-ESI-QToF) method in negative and positive ionization mode

t_R_ (min)	Identified compound	Formula	Mass experimental	Mass calculated	Error (ppm)	[M-H]^−^ m/z	[M + H]^+^ m/z	MS-MS fragments	Presence in flower/leaves/both	References
4.90	Glucaric acid	C_6_H_10_O_8_	210.0376	210.0376	0.13	209.0303		115.0034	Both	Fernández-Poyatos et al. 2019
5.08	Gluconic acid	C_6_H_12_O_7_	196.0583	196.0583	0.09	195.0510		159.0270, 129.0191, 105.0163	Both	Felipe et al. 2014
6.02	Fumaric acid	C_4_H_4_O_4_	116.0110	116.0111	1.36	115.0036		105.2635, 74.6429	Leaves	Sinha et al. 1981; Nandi and Lyndem 2016
6.15	Quinic acid	C_7_H_12_O_6_	192.0634	192.0631	-1.27	191.0541		111.0078, 57.0343, 87.008	Both	Llorent-Martínez et al. 2015
31.51	p-Coumaric acid glucoside	C_15_H_18_O_8_	326.1001	326.0997	-1.31	325.0927		163.0397, 119.0500	Leaves	Simirgiotis et al. 2015; Saha et al. 2018
48.20	Apigenin glucuronide	C_21_H_18_O_11_	446.0849	446.0846	-0.71	445.0775		269.0456, 175.0228	Flower	Uddin et al. 2020; Srivastava et al. 2021
54.82	Acacetin glucuronide	C_22_H_20_O_11_	460.1006	460.1021	3.48	459.2945		283.0640, 268.0405, 113.0253	Flower	Sinha et al. 1981; Uddin et al. 2020; Srivastava et al. 2021
58.72	Apigenin	C_15_H_10_O_5_	270.0528	270.0533	1.9	269.0463		151.0040, 117.0350	Flower	Srivastava et al. 2021
8.50	Caffeic acid	C_9_H_8_O_4_	180.0422	180.0420	-1.4		181.0493	108.0305, 82.0494, 65.0447	Leaves	Dantas et al. 2015; Saha et al. 2018
48.69	Arbutin	C_12_H_16_O_7_	272.0896	272.0891	-1.83		273.0963	255.1739, 240.1513, 225.1267	Both	Chen et al. 2014

In the *C. viscosum* flower extract we found common flavonoids or derivatives also present in other plants, including other *Clerodendrum* species. Apigenin, apigenin glucuronide and acacetin glucuronide are chemical constituents also obtained from *C. infortunatum* (= *C. viscosum*) (Uddin et al. 2020; Srivastava et al. 2021) (Table 2). Apigenin is a common dietary flavonoid with anti-inflammatory, antioxidant or anti-bacterial properties (Yan et al. 2017). Indeed, it has been recently reported for being efficacious for the control of hematophagous mosquitoes *Culex quinquefasciatus*, causing significant damage in the midgut (Samuel et al. 2023). Acacetin is a flavone that has been mostly studied for its benefits on cardiovascular pathologies (Han et al. 2020; Liu et al. 2021; Wu et al. 2021).

Some molecules were found in both flowers and leaves of *C. viscosum* extracts via HPLC-ESI-QToF analysis (Table 2). Quinic acid is an abundant compound in different plant sources and present in coffee, cranberries or kiwifruit (Coppola et al. 1978; Heatherbell et al. 1980; Olthof et al. 2001) with clinical applications due to its capacity to modulate in vivo pancreatic beta-cell function and insulin secretion in mice (Heikkilä et al. 2019). Moreover, quinic acid protects plants from damage caused by invasive western flower thrips, *Frankliniella occidentalis*, becoming this cyclic polyol a potential application as a biocontrol agent in order to manage thrips (Liu et al. 2022).

The organic acids, glucaric acid and gluconic acid, found in both samples have been more studied in chemical industrial applications and for microbial interaction and growth, respectively (Nieto-Peñalver et al. 2014; Zhang et al. 2020). Interestingly, gluconic acid is produced by insect gut bacteria (Khan et al. 2020) and thus as shown in tick microbiota, could be targeted to reduce organism fitness (Mateos-Hernández et al. 2020).

The phenolic compound arbutin was found in both samples. This product is a derivative from the phenolic compound hydroquinone that has been widely found in leaves and used for the development of green products to control parasite infection in plants such as nematodes in tomato plants (Oliveira et al. 2019). This repellent capacity is supported by findings of natural isolates of Enterobacteriaceae that actively hydrolyse plant-derived compounds such as arbutin, to scape predation by bacterivorous amoeba and nematodes (Sonowal et al. 2013), and *Lactobacillus* present in tick and insect microbiota, also characterized as probiotic interventions produce acid from arbutin (Li and Gu 2022; Zhang et al. 2022; Gupta et al. 2023).

Relevant for the objective of this study, the compounds caffeic acid, fumaric acid and p-coumaric acid glucoside were found only in *C. viscosum* leaf extract with repellent activity shown in our assay against *I. ricinus* ticks (Fig. 2; Table 2). It has been reported that some flavonoids and phenolic active compounds are natural biomolecules that exhibit tyrosinase inhibitory activity, being this function essential for some insect and parasite metamorphosis (Panzuto et al. 2002; Kubo et al. 2003; Pantoja Pulido et al. 2017). Interestingly, caffeic acid and derivatives, isolated from natural resources such as leaves, seem to be the most studied compounds in terms of tick-borne diseases due to their antiviral effects, strong antioxidant activity and insect growth control (Pantoja Pulido et al. 2017). Caffeic acid and its derivatives have been found in plants of the genus *Pulicaria*, used in traditional medicine as insect deterrent (Malarz et al. 2023). This polyphenol has been deeply studied for its inhibitory effect on replication of hepatitis B and C virus (Wang et al. 2009; Shen et al. 2013). Apart for being responsible for aroma or colour, in East Asia it has been examined for its robust inhibitory effect against severe fever with thrombocytopenia syndrome (SFTS) (Ogawa et al. 2018), an emerging tick-borne pathology transmitted to humans due to several bites of tick species such as *Haemaphysalis longicornis* and *Amblyomma testudinarium* (Yun et al. 2014).

In case of fumaric acid, no data are available related to a specific protective tick-infection effect. Nevertheless, it has been elucidated for this organic acid to have powerful antimicrobial effect properties against several foodborne pathogens (Ramos et al. 2013; Salaheen et al. 2014; Park et al. 2016). Moreover, biosynthesis of functional metabolites such as fumaric acid is associated with survival and development at extreme high and low temperatures in Diptera such as *Sitodiplosis mosellana* (Huang et al. 2022), a process that may be also present during tick developmental and pathogen transmission dynamics (Gray et al. 2016).

*Clerodendrum viscosum* has been reported for its antimicrobial activity on bacteria and fungi (Rajakaruna et al. 2002; Oly et al. 2011; Amin et al. 2012), a capacity that might be associated to this type of natural compounds found on leaves. Antioxidant, anti-parasite and anti-mutagenesis activities have been also associated to p-coumarics phenolic acids due to the tyrosinase inhibitor capacity, also found in leave extract conjugated as p-coumaric acid glucoside (Pei et al. 2016; Oliveira et al. 2019; Lopes et al. 2020).

Other studies have reported the acaricidal activity against *Rhipicephalus* (*Boophilus*) sp. of derived-phytochemicals from traditional African plants such as terpenes, flavonoids and phenolic compounds, similar to those elucidated in this study (Alain et al. 2022). These secondary metabolites might also be involved in this function against other ticks. A previous GC-MS analysis of *C. viscosum* methanol extract also revealed the presence of compounds with antioxidant and/or antimicrobial activity (Ghosh et al. 2015). The identification in this study of new compounds present in a tick-repellent extract of leaves contribute to fully characterize *C. viscosum* plants together with those already found by other methodologies that may act in combination.

## Conclusions

The results from this study provided evidence of in vivo function of *C. viscosum* as a tick repellent in accordance with a scientific-based traditional medicine of Nepali culture. These results identified potential target bioactive compounds that might be used as natural chemical combinations for the design of protective drugs or repellents in tick-rich environments, especially in rural areas in which health and economic resources are limited. Future research is needed related to concentrations and toxicity properties of *C. viscosum* extracts and their principal components. This study provided in vivo demonstration of repellent activity from a leave extract and elucidated additional information of the natural composition to support indigenous medicine and arise further exploration of new and safe biological products and applications for the development of tick repellents based on natural sources.

## Electronic supplementary material

Below is the link to the electronic supplementary material.

Supplementary Material 1

Supplementary Material 2
